# Violation of the *T*^−1^ Relationship in the Lattice Thermal Conductivity of Mg_3_Sb_2_ with Locally Asymmetric Vibrations

**DOI:** 10.34133/2020/4589786

**Published:** 2020-11-30

**Authors:** Yifan Zhu, Yi Xia, Yancheng Wang, Ye Sheng, Jiong Yang, Chenguang Fu, Airan Li, Tiejun Zhu, Jun Luo, Christopher Wolverton, G. Jeffrey Snyder, Jianjun Liu, Wenqing Zhang

**Affiliations:** ^1^State Key Laboratory of High Performance Ceramics and Superfine Microstructure, Shanghai Institute of Ceramics, Chinese Academy of Sciences, Shanghai 200050, China; ^2^Center of Materials Science and Optoelectronics Engineering, University of Chinese Academy of Sciences, Beijing 100049, China; ^3^Materials Genome Institute, Shanghai University, Shanghai 200444, China; ^4^Department of Materials Science and Engineering, Northwestern University, IL 60208, USA; ^5^Max Planck Institute for Chemical Physics of Solids, Dresden 01187, Germany; ^6^State Key Laboratory of Silicon Materials, School of Materials Science and Engineering, Zhejiang University, Hangzhou 310027, China; ^7^School of Materials Science and Engineering, Shanghai University, Shanghai 200444, China; ^8^Department of Physics and Shenzhen Institute for Quantum Science & Engineering, Southern University of Science and Technology, Shenzhen 518055, China; ^9^Guangdong Provincial Key Lab for Computational Science and Materials Design and Shenzhen Municipal Key Lab for Advanced Quantum Materials and Devices, Southern University of Science and Technology, Shenzhen 518055, China

## Abstract

Most crystalline materials follow the guidelines of *T*^−1^ temperature-dependent lattice thermal conductivity (*κ*_*L*_) at elevated temperatures. Here, we observe a weak temperature dependence of *κ*_*L*_ in Mg_3_Sb_2_, *T*^−0.48^ from theory and *T*^−0.57^ from measurements, based on a comprehensive study combining *ab initio* molecular dynamics calculations and experimental measurements on single crystal Mg_3_Sb_2_. These results can be understood in terms of the so-called “phonon renormalization” effects due to the strong temperature dependence of the interatomic force constants (IFCs). The increasing temperature leads to the frequency upshifting for those low-frequency phonons dominating heat transport, and more importantly, the phonon-phonon interactions are weakened. In-depth analysis reveals that the phenomenon is closely related to the temperature-induced asymmetric movements of Mg atoms within MgSb_4_ tetrahedron. With increasing temperature, these Mg atoms tend to locate at the areas with relatively low force in the force profile, leading to reduced effective 3^rd^-order IFCs. The locally asymmetrical atomic movements at elevated temperatures can be further treated as an indicator of temperature-induced variations of IFCs and thus relatively strong phonon renormalization. The present work sheds light on the fundamental origins of anomalous temperature dependence of *κ*_*L*_ in thermoelectrics.

## 1. Introduction

The study of thermal conductivity has been driven by the increasing concern on both intriguing physical phenomena and powerful technical applications [1]. The thermal conductivity in most crystalline materials consists of the lattice thermal conductivity (*κ*_*L*_) and the electronic component (*κ*_*e*_), which is related to the electrical conductivity (*σ*) through the Wiedemann-Franz law, *κ*_*e*_ = *LσT*, where *L* is the Lorenz number and *T* is the temperature. High thermal conductivity materials such as diamond and silicon are investigated in the area of thermal management of electronics. Low thermal conductivity materials like Zintl phases [2, 3], skutterudites [4, 5], half-Heuslers [6, 7], and materials with chemical bond hierarchy [8–10] are widely used in high-performance thermoelectric energy conversion.

Phonons are quantized collective atomic vibrations. The heat capacity *C*_*v*_, the phonon velocity *v*_*g*_, and the relaxation time *τ* make contributions for *κ*_*L*_ [1] in the phonon gas model according to

(1) $$\begin{array}{c} \kappa_{L}=\frac{1}{NV}\underset{\lambda }{\sum }C_{v}\left(\omega_{\lambda }\right)v_{g}^{2}\left(\omega_{\lambda }\right)\tau \left(\omega_{\lambda }\right), \end{array}$$

where *N* is the number of atoms and *V* is the volume of the cell. The phonon relaxation time *τ* covers the influences from all different scattering mechanisms, including intrinsic mechanisms phonon-phonon interactions and electron-phonon interactions, as well as extrinsic ones, e.g., point defect and grain boundary. The anharmonic three-phonon scattering rate [11] can be expressed as

(2) $$\begin{array}{c} \tau_{\lambda }^{-1}\left(\omega \right)=\frac{\hbar \pi }{8}\underset{\lambda^{\prime }\lambda^{\prime \prime }}{\sum }{\left|\Phi_{\lambda \lambda^{\prime }\lambda^{\prime \prime }}\right|}^{2}\left[\left(n_{\lambda^{\prime }}+n_{\lambda^{\prime \prime }}+1\right)\delta \left(\omega -\omega_{\lambda^{\prime }}-\omega_{\lambda^{\prime \prime }}\right)+2\left(n_{\lambda^{\prime }}-n_{\lambda^{\prime \prime }}\right)\delta \left(\omega -\omega_{\lambda^{\prime }}+\omega_{\lambda^{\prime \prime }}\right)\right]\Delta_{qq^{\prime }q^{\prime \prime }}=2\Gamma_{\lambda }\left(\omega \right), \end{array}$$

where *n*_*λ*_ is the occupation number of the *λ* phonon mode under the Bose-Einstein distribution. Δ_*qq*′*q*^″^_ is the conservation of the momentum. The strength of three-phonon interactions *Φ*_*λλ*′*λ*^″^_ is obtained by

(3) $$\begin{array}{c} \Phi_{\lambda \lambda^{\prime }\lambda^{\prime \prime }}=\underset{ijk}{\sum }\underset{\alpha \beta \gamma }{\sum }\frac{e^{\alpha }\left(i,\lambda \right)e^{\beta }\left(j,\lambda \right)e^{\gamma }\left(k,\lambda \right)}{\sqrt{m_{i}m_{j}m_{k}}\sqrt{\omega_{\lambda }\omega_{\lambda^{\prime }}\omega_{\lambda^{\prime \prime }}}}\Phi_{ijk}^{\alpha \beta \gamma }e^{i\left(q∙r_{i}+q^{\prime }∙r_{j}+q^{\prime \prime }∙r_{k}\right)}, \end{array}$$

where *Φ*_*ijk*_^*αβγ*^ represents (3^rd^-order) anharmonic interatomic force constant (IFC) tensor, *ijk* denote the indexes of atoms, *αβγ* are the Cartesian components, and *e*^*α*^(*i*, *λ*) is the eigenvector for atom *i* at the *λ* phonon mode. Above the Debye temperature, all phonon modes are excited, and the Bose-Einstein distribution is proportional to the temperature, so that the lattice thermal conductivity in crystalline materials exhibits a *T*^−1^ relationship at high temperatures.

Another precondition of the *T*^−1^ relationship of *κ*_*L*_ is the temperature-independent harmonic and anharmonic IFCs. It is generally applicable in high *κ*_*L*_ materials [12] at high temperatures. The well-known Slack *κ*_*L*_ model is just based on the fixed IFCs and high temperature limit to obtain the *T*^−1^ relationship. Some other studies, using phonon dispersions to predict *κ*_*L*_ of solids, also assume constant IFCs in their works [13, 14]. However, for some low thermal conductivity crystalline materials, the typical *T*^−1^ relationship, even at high temperatures without other extrinsic scattering mechanisms, is often violated. For these materials, the conventional first-principles theory for thermal conductivities usually fails since it is based on the lowest-order perturbative treatment and insufficient for strongly anharmonic materials. There are several possible effects existing in these materials, which change the temperature dependence of *κ*_*L*_s, including the alteration of phonon scattering phase space [15], fourth-order phonon scattering [16], and off-diagonal terms in heat current operator [17]. Revealing the fundamental origin of the deviation on *T*^−1^ relationship in each case compound will advance the understanding of the effect of high-order IFCs on thermal transport.

The compound interested in this work is Mg_3_Sb_2_, one of the binary layered compounds. It has attracted increasing attention as a novel n-type thermoelectric material near room temperature, due to an inherent conduction band minimum with six conducting carrier pockets and a tunable bandgap using the alloy effect reported by several independent groups [18–22]. The excess Mg, compensating Mg vacancies [23], is essential to achieve n-type Mg_3_Sb_2_-based materials, with a peak *ZT* up to 1.6 [24]. On the other hand, low *κ*_*L*_ of Mg_3_Sb_2_ is also one of the significant factors for it to become applicable in thermoelectrics. Shearing of adjacent layers [25], large mode Grüneisen parameters [25], weak chemical bonding [21], and weak anharmonic potential wells [21] have been proposed to prove strong anharmonic effects in Mg_3_Sb_2_, which lead to low *κ*_*L*_.

As shown in Fig. S1 [26–28], the experimental *κ*_*L*_s of pristine Mg_3_Sb_2_ clearly have a weaker temperature dependence, usually demonstrating *T*^−0.6^ ~ *T*^−0.9^. The low carrier concentration of pristine Mg_3_Sb_2_ prohibits a strong electron-phonon scattering on *κ*_*L*_, which is the origin of weaker temperature dependence of *κ*_*L*_s in other materials [29–31]. As will be shown later in this work, single crystalline Mg_3_Sb_2_ shows a similar behavior as of the polycrystalline sample for the weaker temperature dependence of *κ*_*L*_, which is the research focus of this work. Our theoretical *κ*_*L*_ of Mg_3_Sb_2_ shows a weaker temperature dependence as of *T*^−0.48^ along both *x* and *z* directions. This result is verified by our experimental data on single crystal Mg_3_Sb_2_. Detailed analysis reveals that some of the phonon vibrational modes at a low-frequency range become higher in frequency with increasing temperatures, due to the influence of the higher-order (particularly, 4^th^-order) IFCs and the renormalization of the harmonic phonons. More importantly, we reveal that the effective 3^rd^-order IFCs become weaker with increasing temperatures, which is the main reason for the weak temperature dependence of *κ*_*L*_ in Mg_3_Sb_2_. The surprising variation of 3^rd^-order IFCs is due to the asymmetric displacements of intralayer Mg atoms, which play a decisive role in determining the lattice anharmonicity. The present work advances the understanding of the temperature-dependent *κ*_*L*_ beyond the classic three-phonon diagram in thermoelectric materials.

## 2. Result and Discussion

**Figure 1:**
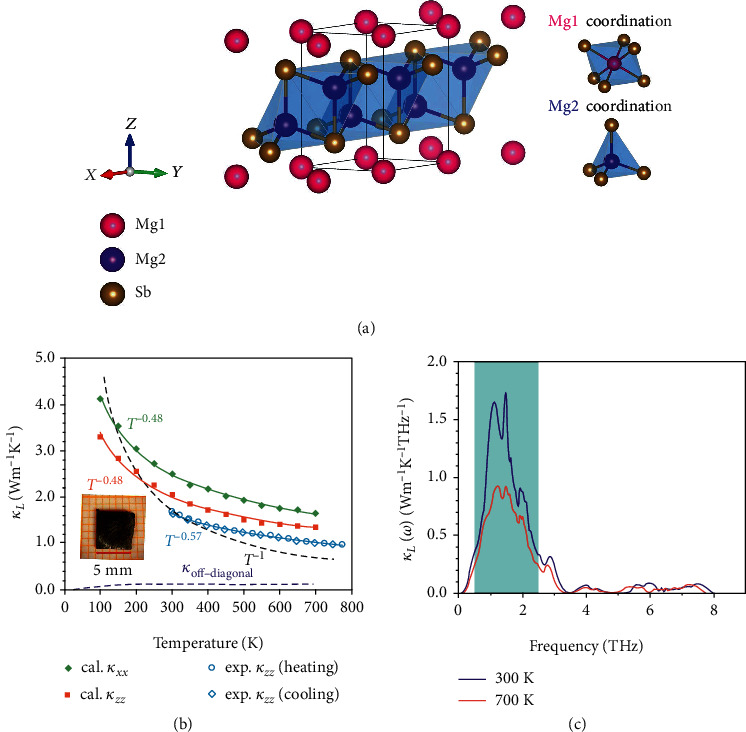
(a) The crystal structure of Mg_3_Sb_2_ (generally expressed as MgMg_2_Sb_2_) with space group $P\bar{3}m1$. (b) The theoretical (solid symbols) and experimental (hollow symbols) temperature-dependent *κ*_*L*_ in Mg_3_Sb_2_. The black dashed line represents the typical *T*^−1^ relationship. The purple dashed line indicates the off-diagonal thermal conductivity for Mg_3_Sb_2_. The inset image is a Mg_3_Sb_2_ single crystal sample, grown by the self-flux method with Sb as flux. (c) Frequency-dependent *κ*_*L*_ in Mg_3_Sb_2_ at 300 K and 700 K.

The pristine Mg_3_Sb_2_, with only five atoms per primitive cell, is described as the Zintl-type structure, combining the ionic Mg^2+^ layer (with the Mg atoms labeled as Mg1) and the covalent (although the nature of the bonding is debatable [21]) [Mg_2_Sb_2_]^2-^ layer (with the Mg atoms labeled as Mg2) as shown in Figure 1(a). Mg1 is on the octahedron site, while Mg2 is on the tetrahedron site. There are two main features in the temperature-dependent *κ*_*L*_ calculations for Mg_3_Sb_2_. One is that we consider the thermal expansion (Fig. S3 (a), discussed later), the other is that the effective 2^nd^-order and 3^rd^-order IFCs are extracted from *ab initio* molecular dynamics (AIMD) under the corresponding volume at finite temperatures, and *κ*_*L*_s can be obtained (Figure 1(b)) using the temperature-dependent effective potential (TDEP) method [32, 33]. The calculations consider all effects from finite temperatures, so we call that finite temperature method (FTM). The calculated *κ*_*L*_ has values of 2.05 W m^−1^ K^−1^ and 2.50 W m^−1^ K^−1^ along *z* and *x* directions at 300 K, respectively. The temperature dependence of *κ*_*L*_ follows *T*^−0.48^, along both directions. In order to explore whether the off-diagonal contribution would affect the temperature dependence, we employed a unified formalism recently developed by Simoncelli et al. [17]. As shown in Figure 1(b), we find that magnitude of the off-diagonal thermal conductivity is very small over the entire temperature range and therefore has a negligible impact on the temperature dependence of *κ*_*L*_ in Mg_3_Sb_2_. Interestingly, in our FTM results, *κ*_*L*_ along the *z* direction is around 20% lower than that along the *x* direction. This is due to the differences in the group velocities (Fig. S2 (a)), i.e., the average of all modes *v*_*x*_ = 430 m/s and *v*_*z*_ = 340 m/s at 300 K. Previous reports in Reference [21] mentioned the nearly isotropic heat conduction of Mg_3_Sb_2_, with a room temperature value of approximately 1.1 W m^−1^ K^−1^. Their isotropic properties come from the almost identical group velocities in different axes, *v*_*x*_ = 470 m/s and *v*_*z*_ = 430 m/s (Fig. S2 (b)). On the other hand, the lowered *κ*_*L*_s in the frozen phonon method [21] are caused by the overestimated Grüneisen parameters, which will be discussed later.

To establish a convincing comparison between theory and experiment, we synthesized the Mg_3_Sb_2_ single crystal sample (the inset image, 5 mm × 5 mm sheet) to eliminate the impacts of defects or grain boundaries on the thermal conductivity [34] as far as possible. *κ*_*L*_ of the Mg_3_Sb_2_ single crystal above 300 K is measured and also shown in Figure 1(b). The experimental heating and cooling curves are on top of each other, indicating that the single crystal Mg_3_Sb_2_ is thermally stable below 773 K, the highest temperature experimentally performed. Notably, from our previous study [35], Mg_3_Sb_2_ single crystals grown from the Sb flux method exhibit very high electrical resistivity (several *Ω*m at room temperature but can be made conducting with good *ZT* by annealing in Mg-rich environment [36]), and thus, the electronic component of the thermal conductivity *κ*_*e*_ is negligible. *κ* is thus almost contributed by the lattice component, i.e., *κ*_*L*_ ≈ *κ*. In the following part, we simply use *κ*_*L*_ instead of *κ* facilitating the comparative discussion with the theoretically calculated *κ*_*L*_. The experimental *κ*_*L*_ for Mg_3_Sb_2_ single crystal is measured along the *z* direction, and the value is around 0.3 W m^−1^ K^−1^ lower than our theoretical value over the entire temperature range. The small deviation might come from possible measurement uncertainty owing to the small size of the single crystal and instrument accuracy. It is noted that our experimental *κ*_*L*_ of Mg_3_Sb_2_ is also weakly temperature-dependent, following approximately *T*^−0.57^ relationship, consistent with our calculations.

Our FTM considers the accurate volume expansion at finite temperatures. The quasiharmonic approximation [37] with two solutions of IFCs is presented in Fig. S3 (a). One is the frozen phonon method; the other is FTM with temperature 100 K. It is found that the slope of the volume-temperature curve from FTM is more consistent with the high-temperature X-ray diffraction results, especially at high temperatures, despite the general overestimation of lattice constants due to Perdew-Burke-Ernzerhof (PBE) functional [38]. The corresponding experimental lattice parameters of Mg_3_Sb_2_ can be found in Fig. S3 (b). The calculated volumetric thermal expansion coefficient for Mg_3_Sb_2_ is 4.7 × 10^−5^ K^−1^ (4.2 × 10^−5^ K^−1^) at 300 K (700 K), which is close to Bi_2_Te_3_ (5.2 × 10^−5^ K^−1^, 200-300 K [39]), slightly higher than PbTe (2.0 × 10^−5^ K^−1^, 300 K [40]), and distinctly higher than those high thermal conductivity materials, like diamond (0.3 × 10^−5^ K^−1^, 300 K [41]) and silicon (0.8 × 10^−5^ K^−1^, 300 K [42]). The relatively large thermal expansion coefficient indicates the considerable anharmonicity of Mg_3_Sb_2_ like other low *κ*_*L*_ thermoelectric materials, such as Bi_2_Te_3_ [43] and PbTe [44].

Figure 1(c) displays the frequency-dependent *κ*_*L*_ at 300 K and 700 K. Note that *κ*_*L*_s in these two plots are the averaged ones along the three Cartesian axes. We see that phonons which dominate the *κ*_*L*_s generally have frequencies below 3.5 THz and mostly are between 0.5 and 2.5 THz (Figure 1(c)) at both temperatures. Therefore, the following discussion will focus on the low-frequency region. The cumulative *κ*_*L*_s in Fig. S4 for Mg_3_Sb_2_ as a function of the mean free path (MFP) show that phonon modes in Mg_3_Sb_2_ have a maximum MFP of *κ*_*L*_ around 200 nm at both temperatures, and half of the *κ*_*L*_s are from those modes with MFP less than 10 nm. These are useful for the design of nanostructures in Mg_3_Sb_2_ in order to further suppress *κ*_*L*_.

**Figure 2:**
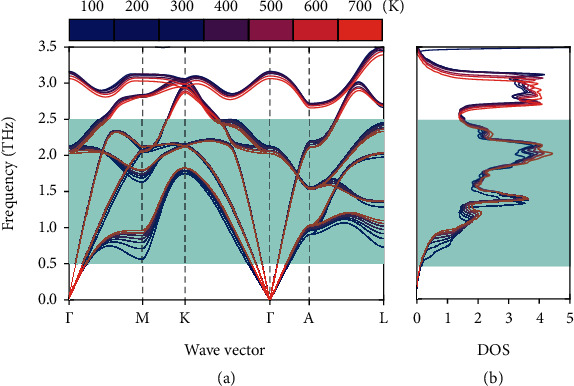
(a) Calculated temperature-dependent phonon dispersions of Mg_3_Sb_2_ from *T* = 100 K to 700 K. (b) The total phonon density of states from *T* = 100 K to 700 K. The shade green areas depict phonons that dominate *κ*_*L*_.

Since the possible influence from electron-phonon interaction or off-diagonal contribution has been excluded, here, we focus on the variations of 2^nd^-order and 3^rd^-order IFCs and related physics to explore the origin of the weak temperature-dependent *κ*_*L*_. Due to the fact that the low-frequency phonons dominate *κ*_*L*_ of Mg_3_Sb_2_, temperature-dependent phonon dispersions of Mg_3_Sb_2_ in the low-frequency region are shown in Figure 2(a). (The whole phonon dispersions at 300 K and 700 K are shown in Fig. S5.) It is interesting to note that the phonons in the low-frequency region show diverse temperature dependence. Specifically, the transverse acoustic phonons become harder as temperature increases, especially at the Brillouin zone boundary *M*, *A*, and *L* points. The temperature dependence along Γ‐*A* is contrary to the result in Fig. S6, similar to the result in Reference [25], which only considered the lattice thermal expansion based on the frozen phonon method. The low-lying transverse acoustic branches generally become harder with increasing temperature. For example, the zone-center speed of sound for Mg_3_Sb_2_ along Γ to *A* directions at 300 K (700 K) is 1734 m/s (1909 m/s).

To provide an intuitive physical picture for the phonon hardening, we visualized the atomic vibrations for Mg_3_Sb_2_ corresponding to the low-lying transverse acoustic phonon modes, i.e., *M*, *A*, and *L* points, in Gif S1 (M.gif), Gif S2 (A.gif), and Gif S3 (L.gif). These animations demonstrate that the atomic motions within the [Mg_2_Sb_2_]^2-^ layer dominate the vibrational modes at these **q** points. And since these points are at the zone boundary, Mg2 and Sb in the [Mg_2_Sb_2_]^2-^ layer show the largest phase difference, resulting in the “head-to-head” motions. When temperature increases, the head-to-head motion impedes the vibrations of atoms in the [Mg_2_Sb_2_]^2-^ layer towards the neighboring atoms, thus making the potential energy (Fig. S7) increase more rapidly than the harmonic approximation at *M*, *A*, and *L* points.

The total phonon density of states from *T* = 100 K to 700 K is shown in Figure 2(b). Due to the significant phonon hardening effects with temperature from the low-lying transverse acoustic phonons at Brillouin zone boundary points around 1 THz (Figure 2(a)), the phonon density of states around 1 THz move to a higher frequency with the increased temperature. We verified this conjecture by explicitly computing renormalized phonons using the self-consistent phonon theory that accounts for the first-order correction from quartic IFCs [45] as shown in Fig. S8. The hardening of the low-lying transverse acoustic phonon modes at the *M*, *A*, and *L* points is reproduced, unambiguously confirming the essential role of quartic IFCs in the phonon renormalization.

**Figure 3:**
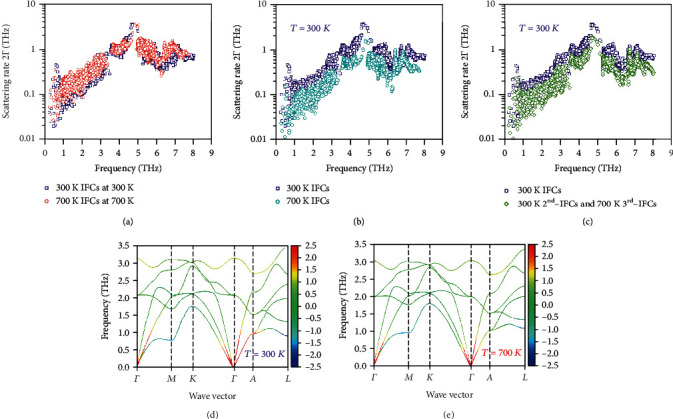
The scattering rates 2Γ from three-phonon interactions (a) at *T* = 300 K and *T* = 700 K using 300 K IFCs and 700 K IFCs, respectively; (b) at *T* = 300 K using, respectively, 300 K IFCs and 700 K IFCs; and (c) at *T* = 300 K with one set of 3^rd^-order IFCs substituted by those obtained at *T* = 700 K. The color-coded mode Grüneisen parameters projected onto the phonon dispersions at (d) *T* = 300 K and (e) *T* = 700 K.

The above analysis mainly focuses on the variations of the 2^nd^-order IFC induced phonon dispersions. On the other hand, by examining the key quantities entering the evaluation of thermal conductivity, namely, the phonon mode-wise heat capacity, group velocity (related to 2^nd^-order IFCs), and phonon relaxation time (inverse phonon scattering rate, related to 2^nd^-order IFCs on the scattering phase space and 3^rd^-order IFCs on the anharmonicity), we use controlled comparisons to clarify the influence of temperature-dependent IFCs on *κ*_*L*_ at 300 K, as shown in Table S1. By separately substituting the 2^nd^-order and 3^rd^-order IFCs, we find that 2^nd^-order IFC related group velocity and scattering phase space have limited impact on *κ*_*L*_, while 3^rd^-order IFC related anharmonicity plays a significant role. These results are distinctly different from previous studies for other low *κ*_*L*_ materials, where only 2^nd^-order IFC related quantities matter [15, 46]. Similarly, we compare the phonon scattering rates (Γ) in Mg_3_Sb_2_ at *T* = 300 K and 700 K, respectively, as shown in Figure 3(a). As expected, the scattering rates at 700 K are higher than those at 300 K. It is because higher temperature results in a larger phonon number (Eq. (2)), thus larger scattering magnitude. However, the scattering rates at 700 K are only slightly larger than those at 300 K, which indicates that the IFCs entering the evaluation of scattering rates might have strong temperature dependence. We examine this conjecture by comparing the scattering rates calculated at 300 K but using temperature-dependent IFCs at 300 K and 700 K, respectively, as shown in Figure 3(b). The significantly smaller scattering rates using IFCs obtained at 700 K clearly reveal the strong temperature dependence of effective IFCs. We, respectively, replace the 2^nd^-order IFCs and the 3^rd^-order IFCs at 700 K with those obtained at 300 K and compare the resulting scattering rates. We find that when the 3^rd^-order IFCs are kept the same while the 2^nd^-order IFCs are from 300 K and 700 K, the resulting scattering rates are largely similar (see Fig. S9). On the other hand, when the 2^rd^-order IFCs are kept the same while the 3^nd^-order IFCs are from 300 K and 700 K, the resulting scattering rates show significantly reduced magnitude for those using the 3^rd^-order IFCs at 700 K (Figure 3(c)). These controlled comparisons confirm that (i) the strong temperature dependence of the 3^rd^-order IFCs plays a decisive role in the unusually weak decay of scattering rates as a function of temperature and (ii) the anharmonicity is reduced at elevated temperatures.

The strength of the anharmonicity at various temperatures can also be estimated from the mode Grüneisen parameters, which are obtained as the logarithmic derivative of the phonon frequencies with respect to the volume, as shown in Figures 3(d) and 3(e). The acoustic phonons show relatively large absolute values of mode-wise Grüneisen parameters (~1.4), while the values of the optical phonons are relatively lower (~0.9), based on the results at 300 K. Specifically, the first phonon modes at *M* and *L* points show negative values, -1.55 and -2.47, respectively, while at the *A* point, the value is positive as of 1.97. The negative Grüneisen parameters at *M* and *L* points mean that the phonon frequency at these points will be increased with lattice expansion and partly contributes to the phonon hardening effects with increasing temperature (volume), as shown in Figure 2(a). On the other hand, the positive Grüneisen parameter at the *A* point cancels some of the phonon renormalization effect, making the frequency change at *A* point the smallest among the three (*M*, *A*, and *L*) points. Furthermore, the Grüneisen parameters from the frozen phonon method (2.0 for acoustic phonons) are larger than those from FTM. This is the reason that previous simulations based on the frozen phonon method underestimated *κ*_*L*_s, approximately 1.1 W m^−1^ K^−1^ at 300 K for Mg_3_Sb_2_. [21] As for the temperature dependence, the Grüneisen parameters at 300 K are higher than those at 700 K (Fig. S10), consistent with the weakened anharmonic phonon scattering in Figure 3(c). In light of this discovery, it is natural to ask what is the physical origin of the strongly temperature-dependent 3^rd^-order IFCs.

**Figure 4:**
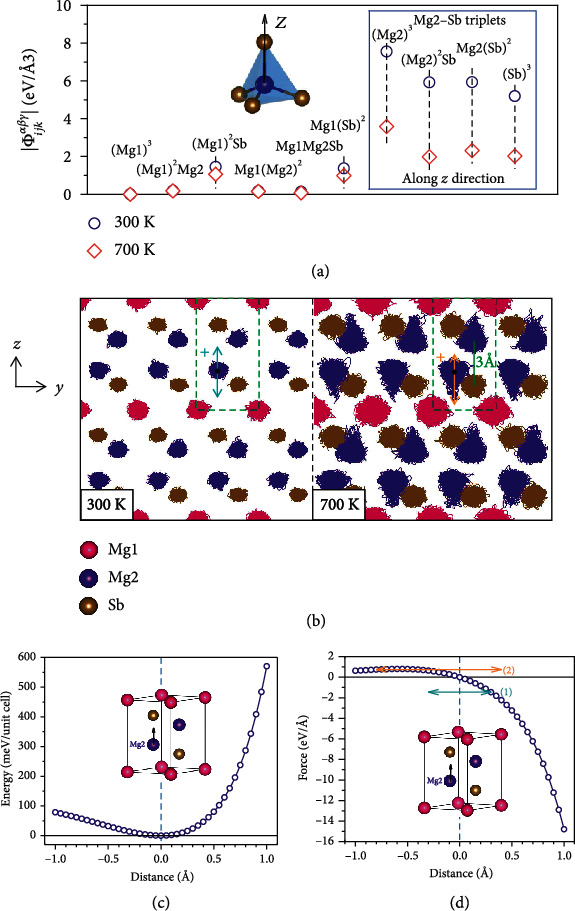
(a) Absolute values of the 3^rd^-order IFCs of various combinations of atoms. (b) The MD trajectories at 300 K and 700 K. The green dashed box is the primitive cell of Mg_3_Sb_2_, and the blue and orange arrows represent the directions we adopted for the following energy and force calculations. (c) Energy-distance curve for Mg2 atoms when Mg2 atom is displaced away from its equilibrium position along the *z*-axis. (d) Same as (c) but for the force-distance curve. Two-headed horizontal arrows in (d) indicate, respectively, the evenly distributed small (1) displacements and unevenly distributed large (2) displacements.

To understand the temperature dependence of the 3^rd^-order IFCs, we compared the 3^rd^-order IFCs for all the triplets at 300 K and 700 K as shown in Figure 4(a). All the triplets listed are along the directions with the largest 3^rd^-order IFCs at 300 K for Mg_3_Sb_2_. Among the various triplets, the ones that involve onsite interactions (e.g., (Mg2)^3^ and (Sb)^3^) or interactions between the neighboring atoms (e.g., (Mg2)^2^Sb and Mg2(Sb)^2^) along the *z* direction have the largest 3^rd^-order IFCs at room temperature. More importantly, these triplets with large 3^rd^-order IFCs also show strong temperature dependence. Specifically, we find that the 3^rd^-order IFC of the onsite interactions of Mg2 atoms along the *z* direction (denoted as (Mg2)^3^_*zzz*_), which show the largest values among all the triplets at 300 K, decreases dramatically from 7.56 eV/Å^3^ at 300 K to 3.58 eV/Å^3^ at 700 K. In the following, we will focus on this set of IFCs to explore the microscopic origin of the reduced IFCs at elevated temperatures.

The MD trajectories at 300 K and 700 K are shown in Figure 4(b). Based on the trajectories, it can be found that the average radii of the atomic displacements at 700 K are much larger than those at 300 K. An unusual feature in Figure 4(b) is that the atomic displacements of all atoms at 300 K are approximately isotropic, while the displacements of Mg2 atoms become strongly anisotropic at 700 K, i.e., a cone-type trajectory. The pointy edge is at the opposite of the neighboring Sb along the *z* direction (assumed to be the positive position w.r.t. Mg2), as shown in Figure 4(b). Quantitatively, according to the probability density as a function of Mg2 displacement along the *z* direction (Fig. S11) at 700 K, there are 18% more points at the negative displacement side, which has a large tail down to over -1 Å. The same number is only 4% at 300 K. This type of Mg2 trajectory at 700 K is due to the restrictions of vibrations from the neighboring Sb atoms, and the IFCs of Mg2 are altered along with the restrictions. The energy and force profiles of Mg2 atom w.r.t. the displacement along the *z* direction, shown in Figures 4(c) and 4(d), help to understand the alteration. As displayed in Figure 4(c), it is found that the energy of the system increases rapidly/slowly when Mg2 atom is displaced along the positive/negative *z* direction. It is thus the asymmetric energy profile along the *z* direction that leads to the asymmetric displacements of Mg2 atoms, and such asymmetry enhances with increasing temperature, consistent with the Boltzmann distribution. The asymmetry is also reflected in the restoring forces on Mg2 atoms, as shown in Figure 4(d). Mg2 atoms at the lower-energy side have much lower forces, and the lowered forces result in lowered 3^rd^-order IFC of onsite (Mg2)^3^_*zzz*_ since the onsite 3^rd^-order IFC is actually the curvature of the force profile. And since the lower-energy side has more sampling at elevated temperatures, i.e., 18% more at 700 K, the 3^rd^-order IFC for the onsite (Mg2)^3^_*zzz*_ is thus lowered by the asymmetry of the force profile.

In fact, the force profile at finite temperatures is much complicated than those presented in Figure 4(d). Fig. S12 (a) shows the force profile along the *z* direction for the same Mg2 atom at 700 K, extracted from the AIMD simulation. The general trend is like Figure 4(d), i.e., positive displacements with larger forces and negative displacements with lowered forces. It is interesting that at some very negative displacements for Mg2 along the *z* direction, the force is close to zero. Fig. S12 (b) shows the local tetrahedron of the Mg2, with the displacement -0.93 Å and the force -0.01 eV/Å. The Mg2 locates at the center of the bottom 3 Sb atoms, and the bond with the top Sb is broken. The close-to-zero force at large negative displacements further contributes to the low (Mg2)^3^_*zzz*_.

The temperature-dependent IFCs, due to the asymmetric displacements of Mg2 atoms, provide useful guidance of exploring materials with *κ*_*L*_ other than *T*^−1^ relationship. The local structure of Mg2 determines an asymmetric potential energy profile, as shown in Figure 4(c); more importantly, the displacements of Mg2 atoms at finite temperatures, such as 700 K, is large enough to have uneven distributions at different sides of the atomic equilibria and finally induce the temperature-dependent IFCs. Inspired by this example, the atomic trajectories from AIMD or thermal ellipsoids can be adopted as indicators for the temperature-dependent IFCs. Strongly asymmetric trajectories or ellipsoids at reachable temperatures will cause the same uneven distributions as the Mg2 in Mg_3_Sb_2_ and thus change the IFCs and *κ*_*L*_ dependence. For example, some fillers in type-I clathrates, such as Ba_8_Ga_16_Ge_30_, Ba atoms show different atomic displacements along different axes at room temperature [47–49], and therefore, *κ*_*L*_s show strong deviations from *T*^−1^, sometimes even glass-like behavior. If one compound has similar asymmetric movement, it may have the temperature dependence violating *T*^−1^ on the lattice thermal transport.

## 3. Conclusions

Layered structure Mg_3_Sb_2_, as a novel thermoelectric material, has achieved increasing interest due to the inherently low *κ*_*L*_. Here, we find that Mg_3_Sb_2_ has weak temperature-dependent *κ*_*L*_ through both simulations and experiments. The weak temperature dependence is due to the renormalizations of both 2^nd^-order IFCs and 3^rd^-order ones, and they can be traced back to the limitations of atomic vibrations at elevated temperatures. For 2^nd^-order IFCs, the covalent [Mg_2_Sb_2_]^2-^ layer exhibits in-plane “head-to-head” motions at the Brillouin zone boundary *M*, *A*, and *L* points for the first transverse acoustic phonons. The phenomenon causes that low-lying transverse acoustic phonons to become hardened. Meanwhile, the asymmetric displacements of Mg2 atoms due to the tetrahedrally bonding nature enhance with increasing temperature, and the effective 3^rd^-order IFCs tend to have a smaller absolute value due to more sampling at the decreased curvature side in the force profile. Both the hardened low-lying phonons and weaker anharmonicity at elevated temperatures contribute to the weak temperature dependence of *κ*_*L*_. And the asymmetric atomic vibrations at finite temperatures can be adopted to explore other materials with temperature-dependent IFCs and abnormal *κ*_*L*_ behavior.

## 4. Methods

### 4.1. Experiment Details

Single crystals of Mg_3_Sb_2_ were grown by using a self-flux method with Sb as flux (details can be found in Reference [35]). Single crystallinity was checked using white-beam backscattering Laue X-ray diffraction at room temperature. Distinct diffraction spots were detected (Fig. S13), matching well with the theoretically simulated pattern based on the $P\bar{3}m1$ space group. The thermal conductivity of Mg_3_Sb_2_ single crystals above room temperature was calculated by using the equation *κ* = *DρC*_*p*_, where *D* is the thermal diffusivity and measured using laser flash analysis instrument (LFA 457, Netzsch), *ρ* is the theoretical density, *C*_*p*_ is the specific heat, calculated using the recommended equation by Agne et al. [50]. Owing to the small size of the single crystal and instrument accuracy, an estimated uncertainty of *κ* is about 10%.

### 4.2. Calculation Details

All first-principle calculations were carried out based on density function theory (DFT) and using the projector-augmented wave (PAW) method, as implemented in the Vienna *ab initio* Simulation Package (VASP) [51]. The thermal expansion properties were calculated with self-consistent quasiharmonic approximation (SC-QHA) [52] with IFCs (obtained by AIMD at 100 K) of Mg_3_Sb_2_ under different volumes. Thus, temperature-dependent *κ*_*L*_s for Mg_3_Sb_2_ can be calculated under the corresponding volume at a given temperature. We performed MD simulation 4 × 4 × 4 supercell, 320 atoms in total from 100 K to 700 K. The MD simulations for Mg_3_Sb_2_ ran for 60 ps with a time of 2 fs, excluding 6 ps to reach the equilibrated state. The Brillouin zone integration was carried out on the Γ point. The plane-wave energy cutoff and energy convergence criterion were set as 200 eV and 10^−4^ eV, respectively, for the MD simulations. The temperature-dependent harmonic and anharmonic IFCs were extracted by TDEP. We processed for heat transport-related properties also with the TDEP code [32, 33].

## Supplementary Materials

Supplementary Materials Fig. S1: experimental thermal conductivity data of polycrystalline samples. Fig. S2: phonon group velocity along three directions for Mg_3_Sb_2_ (a) at 300 K with the finite temperature method and (b) with the frozen phonon method. Fig. S3: (a) the temperature-dependent volumes of the primitive cell of Mg_3_Sb_2_ obtained by the high-temperature X-ray diffraction method, compared to those obtained using frozen phonon and AIMD calculation methods. (b) Lattice parameters are measured by high-temperature X-ray diffraction using Mg_3_Sb_2_ polycrystalline powders covering the range of 300 K to 700 K. Fig. S4: cumulative *κ*_*L*_ versus phonon mean free path for Mg_3_Sb_2_ at 300 K and 700 K. Fig. S5: the whole phonon dispersions of Mg_3_Sb_2_ at 300 K and 700 K. Fig. S6: calculated temperature-dependent phonon dispersions of Mg_3_Sb_2_ from *T* = 100 K to 700 K, only considering the lattice thermal expansion. Fig. S7: the potential energy surface corresponding to the low-lying transverse acoustic phonon modes at the Brillouin zone boundary *M* point (a), *A* point (b), and *L* point (c). Fig. S8: calculated temperature-dependent phonon dispersions of Mg_3_Sb_2_ from *T* = 100 K to 700 K using the self-consistent phonon theory. Fig. S9: the scattering rate 2Γ with 300 K IFCs is compared to 2^nd^-order IFCs, substituted by 700 K, at *T* = 300 K. Fig. S10: frequency-dependent mode Grüneisen parameters using the frozen phonon method, FTM at 300 K and 700 K. Fig. S11: probability density of MD trajectory deviating from equilibrium position at (a) 300 K and (b) 700 K along the *z* direction. Fig. S12: (a) the force profile of Mg2 atom along the *z* direction at 700 K, extracted from molecular dynamics simulation. (b) The local structure of Mg_3_Sb_2_ with the displacement -0.93 Å along the *z* direction and the force -0.01 eV/Å. Fig. S13: (a) Laue diffraction pattern of the studied Mg_3_Sb_2_ single crystal and (b) the theoretically simulated pattern based on $P\bar{3}m1$ space group, matching well with the experimental data. Table S1: the calculated *κ*_*L*_ along *x* and *z* directions at 300 K with temperature-dependent IFCs.
