# Supplementary material for: Violation of the T−1 Relationship in the Lattice Thermal Conductivity of Mg3Sb2 with Locally Asymmetric Vibrations
Source: Research (Wash D C). 2020 Nov 30;2020:4589786. doi: 10.34133/2020/4589786 (PMC7877392; doi:10.34133/2020/4589786)
Supplement: Supplementary Materials — Fig. S1: experimental thermal conductivity data of polycrystalline samples. Fig. S2: phonon group velocity along three directions for Mg3Sb2 (a) at 300 K with the finite temperature method and (b) with the frozen phonon method. Fig. S3: (a) the temperature-dependent volumes of the primitive cell of Mg3Sb2 obtained by the high-temperature X-ray diffraction method, compared to those obtained using frozen phonon and AIMD calculation methods. (b) Lattice parameters are measured by high-temperature X-ray diffraction using Mg3Sb2 polycrystalline powders covering the range of 300 K to 700 K. Fig. S4: cumulative κL versus phonon mean free path for Mg3Sb2 at 300 K and 700 K. Fig. S5: the whole phonon dispersions of Mg3Sb2 at 300 K and 700 K. Fig. S6: calculated temperature-dependent phonon dispersions of Mg3Sb2 from T = 100 K to 700 K, only considering the lattice thermal expansion. Fig. S7: the potential energy surface corresponding to the low-lying transverse acoustic phonon modes at the Brillouin zone boundary M point (a), A point (b), and L point (c). Fig. S8: calculated temperature-dependent phonon dispersions of Mg3Sb2 from T = 100 K to 700 K using the self-consistent phonon theory. Fig. S9: the scattering rate 2Γ with 300 K IFCs is compared to 2nd-order IFCs, substituted by 700 K, at T = 300 K. Fig. S10: frequency-dependent mode Grüneisen parameters using the frozen phonon method, FTM at 300 K and 700 K. Fig. S11: probability density of MD trajectory deviating from equilibrium position at (a) 300 K and (b) 700 K along the z direction. Fig. S12: (a) the force profile of Mg2 atom along the z direction at 700 K, extracted from molecular dynamics simulation. (b) The local structure of Mg3Sb2 with the displacement -0.93 Å along the z direction and the force -0.01 eV/Å. Fig. S13: (a) Laue diffraction pattern of the studied Mg3Sb2 single crystal and (b) the theoretically simulated pattern based on P3¯m1 space group, matching well with the experimental data. Table S1: the cal [file 4589786.f1.zip › 4589786.docx]

**Supplemental Information**

**Violation of the *T*^-1^ relationship in the lattice thermal conductivity of Mg_3_Sb_2_ with locally asymmetric vibrations**

**Yifan Zhu,^1,2,3^ Yi Xia,^4^ Yancheng Wang,^1,2^ Ye Sheng,^3^ Jiong Yang,^3,1*^ Chenguang Fu,^5*^ Airan Li,^6^ Tiejun Zhu,^6^ Jun Luo,^7,3^ Christopher Wolverton,^4^ G. Jeffrey Snyder,^4^ Jianjun Liu,^1,2^ and Wenqing Zhang^8,9,1*^**

*^1^State Key Laboratory of High Performance Ceramics and Superfine Microstructure, Shanghai Institute of Ceramics, Chinese Academy of Sciences, Shanghai 200050, China*

*^2^Center of Materials Science and Optoelectronics Engineering, University of Chinese Academy of Sciences, Beijing 100049, China*

*^3^Materials Genome Institute, Shanghai University, Shanghai 200444, China*

*^4^Department of Materials Science and Engineering, Northwestern University, IL 60208, USA*

*^5^Max Planck Institute for Chemical Physics of Solids, Dresden 01187, Germany*

*^6^State Key Laboratory of Silicon Materials, School of Materials Science and Engineering, Zhejiang University, Hangzhou 310027, China*

*^7^School of Materials Science and Engineering, Shanghai University, Shanghai 200444, China*

*^8^Department of Physics and Shenzhen Institute for Quantum Science & Engineering, Southern University of Science and Technology,* *Shenzhen 518055, China*

*^9^Guangdong Provincial Key Lab for Computational Science and Materials Design, and Shenzhen Municipal Key-Lab for Advanced Quantum Materials and Devices, Southern University of Science and Technology, Shenzhen 518055, China*

Correspondence should be addressed to Jiong Yang, [jiongy@t.shu.edu.cn](mailto:*jiongy@t.shu.edu.cn); Chenguang Fu, [chenguang.fu@cpfs.mpg.de;](mailto:chenguang.fu@cpfs.mpg.de;) Wenqing Zhang, zhangwq@sustech.edu.cn

Yifan Zhu and Yi Xia contributed equally to this work.


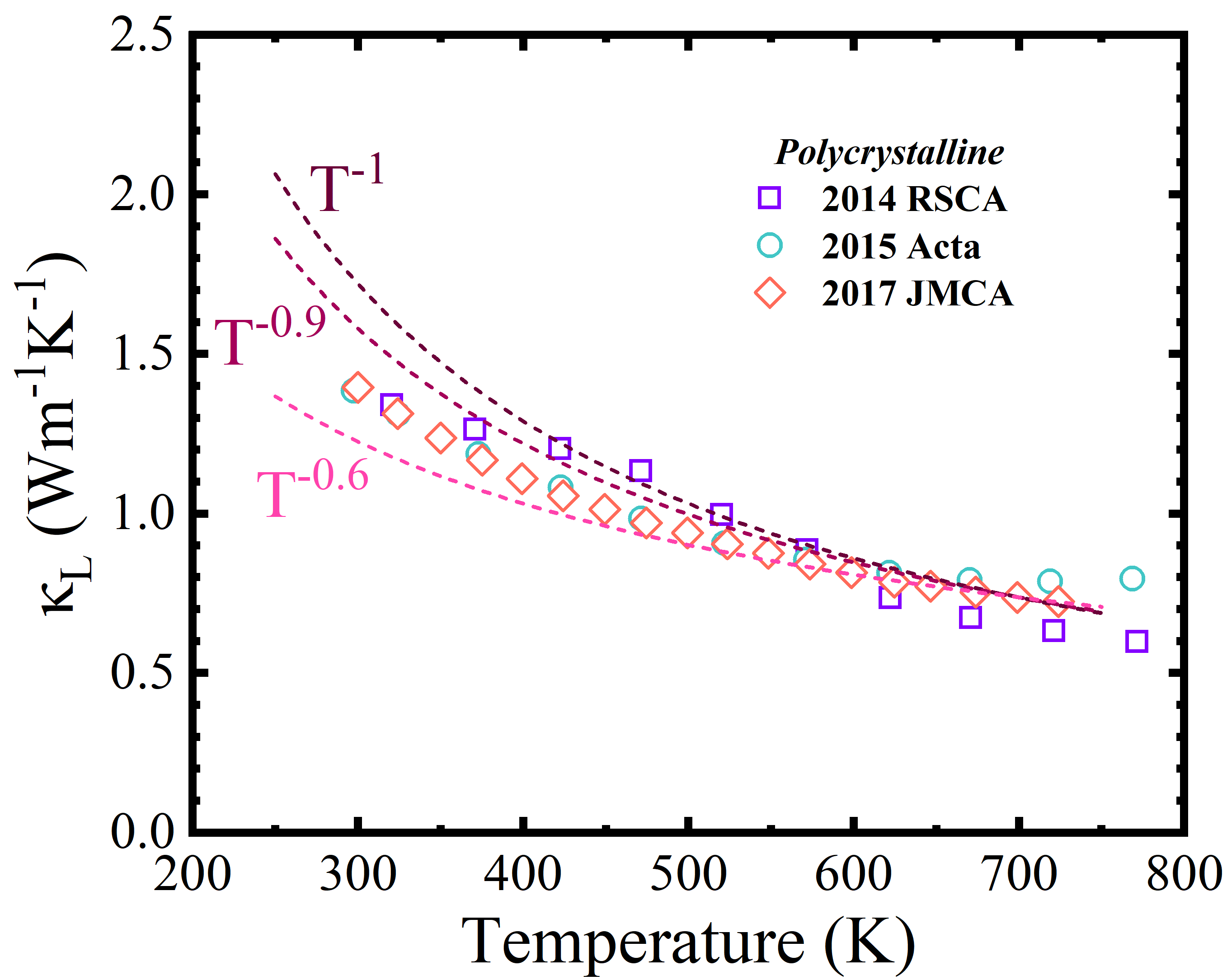


**Fig. S1** Experimental thermal conductivity data of polycrystalline samples[1-3]. The dark brown dash line is *T*^-1^ temperature dependence. The light brown dash line and pink dash line indicate *T*^-0.6^ and *T*^-0.9^, respectively.


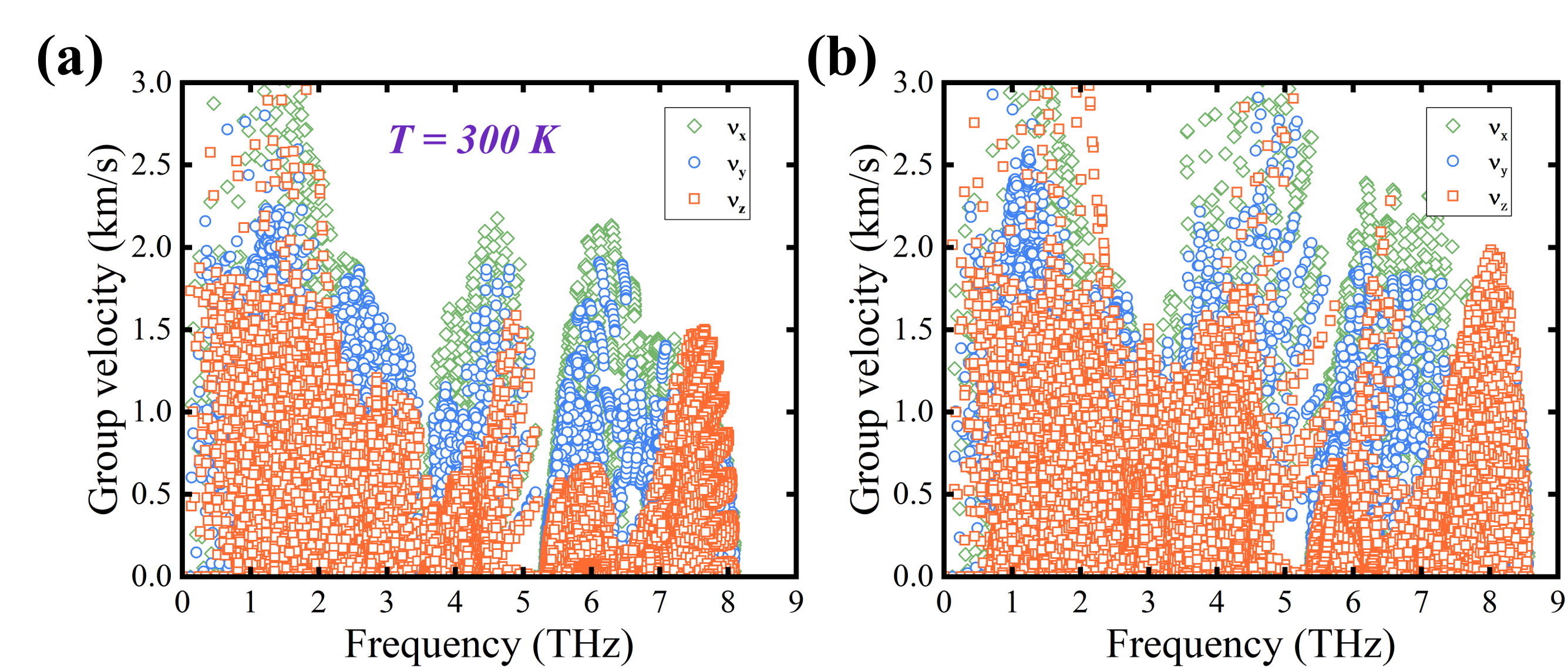


**Fig. S2** Phonon group velocity along three directions for Mg_3_Sb_2_ **(a)** at 300 K with finite temperature method and **(b)** with frozen phonon method[4].


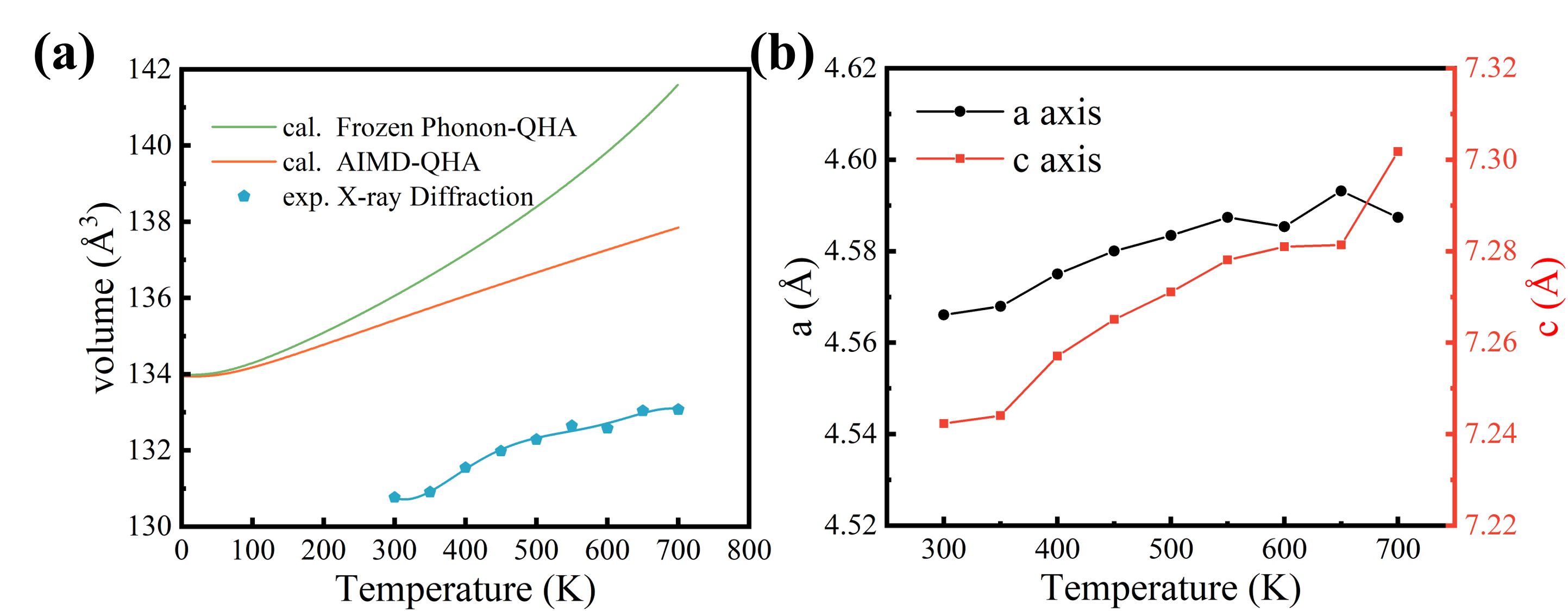


**Fig. S3 (a)** The temperature-dependent volumes of the primitive cell of Mg_3_Sb_2_ obtained by high temperature X-ray diffraction method, compared to those obtained using frozen phonon and AIMD calculation methods. **(b)** Lattice parameters are measured by high temperature X-ray diffraction using Mg_3_Sb_2_ polycrystalline powders cover the range of 300 K to 700 K.

**
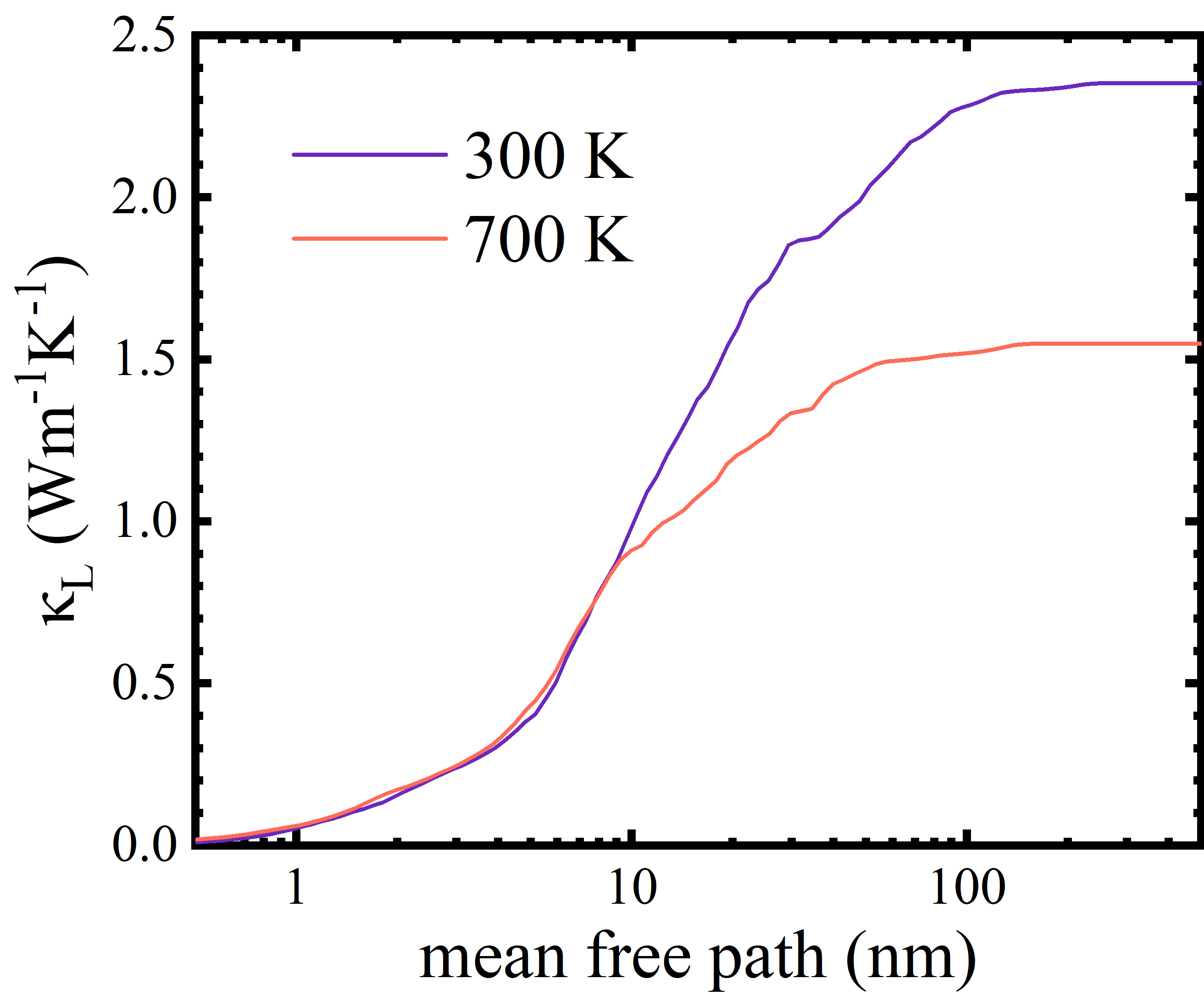
**

**Fig. S4** Cumulative *κ_L_* versus phonon mean free path for Mg_3_Sb_2_ at 300 K and 700 K.


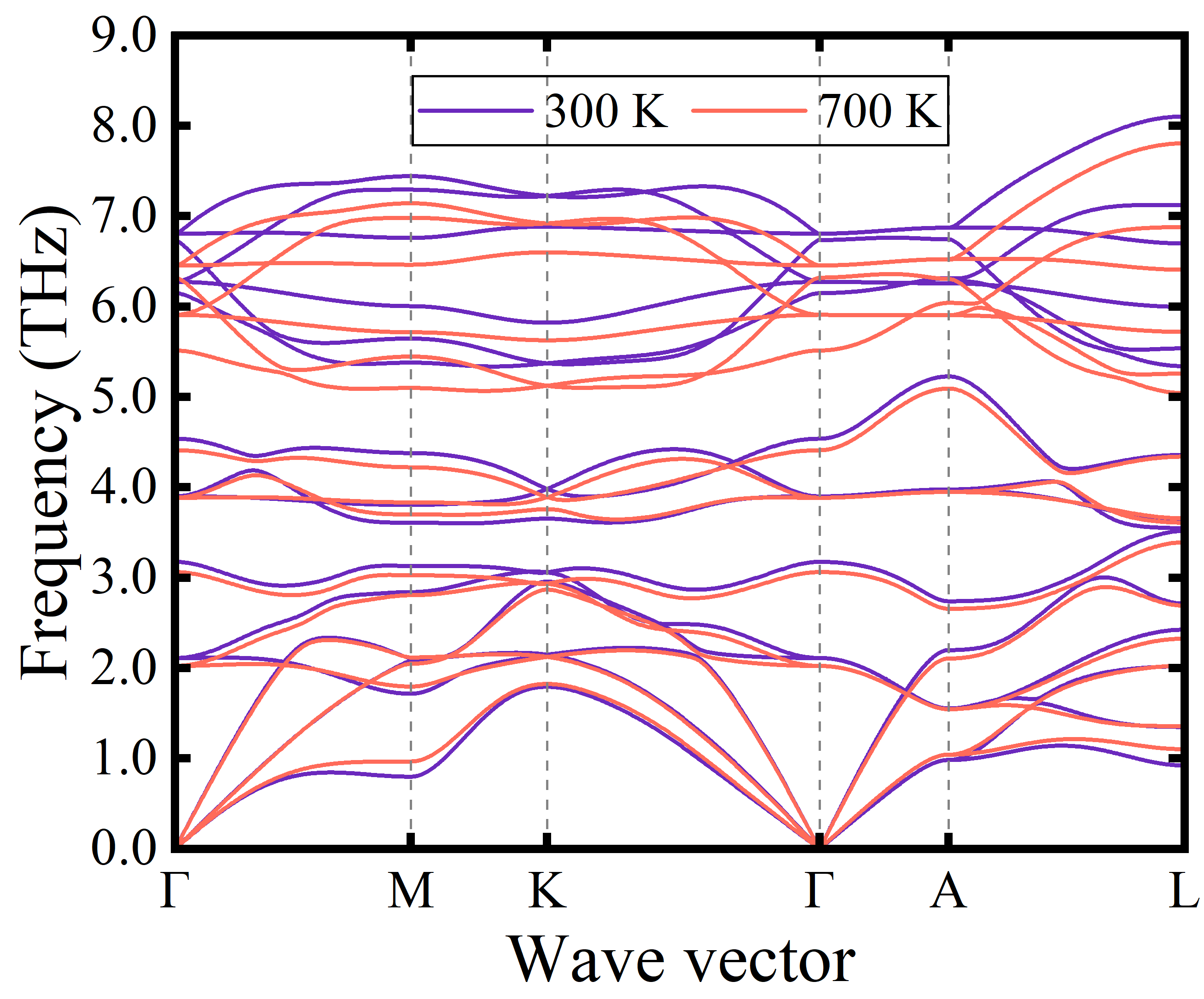


**Fig. S5** The whole phonon dispersions of Mg_3_Sb_2_ at 300 K and 700 K.


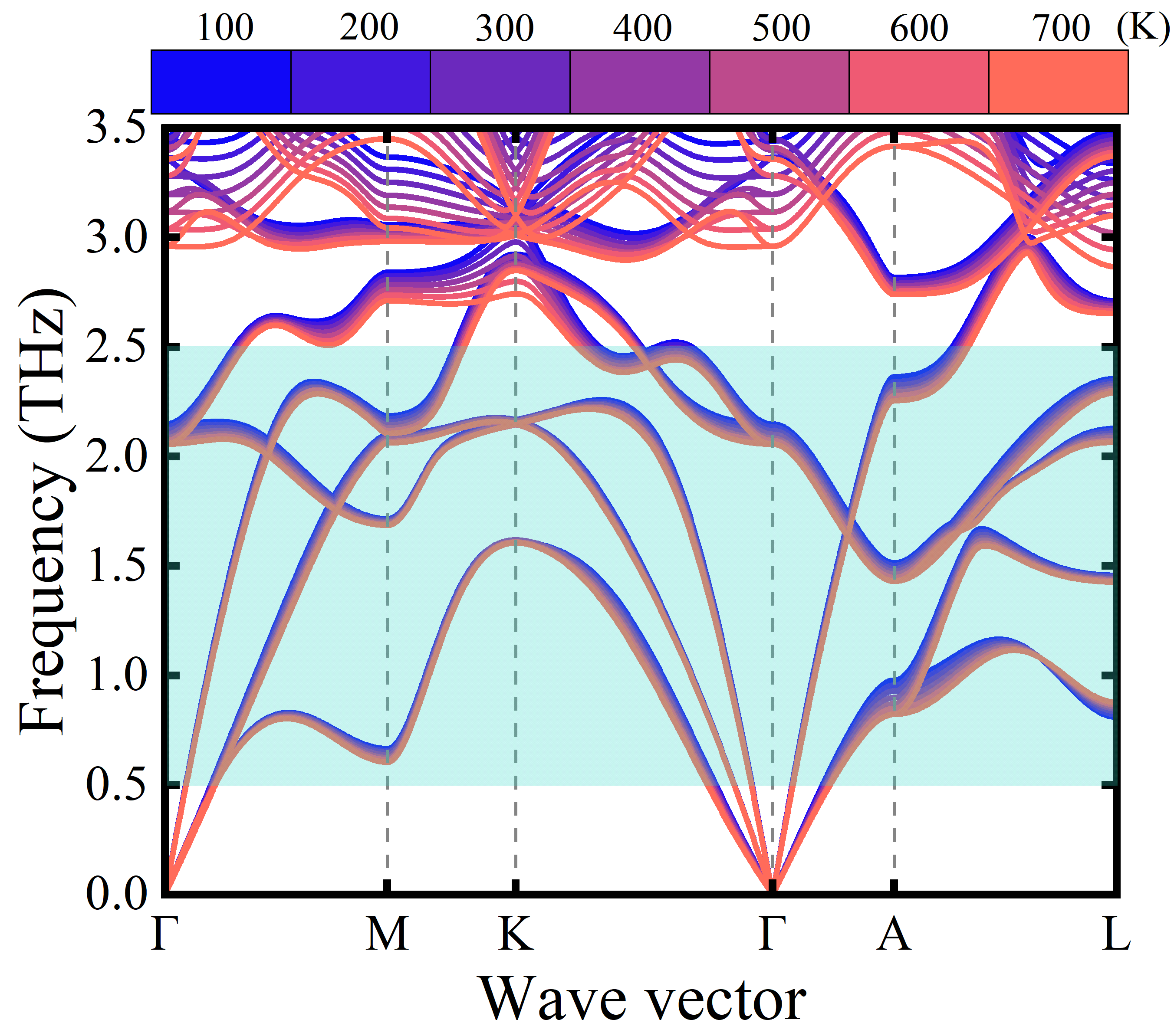


**Fig. S6** Calculated temperature-dependent phonon dispersions of Mg_3_Sb_2_ from *T* = 100 K to 700 K, only considering the lattice thermal expansion.


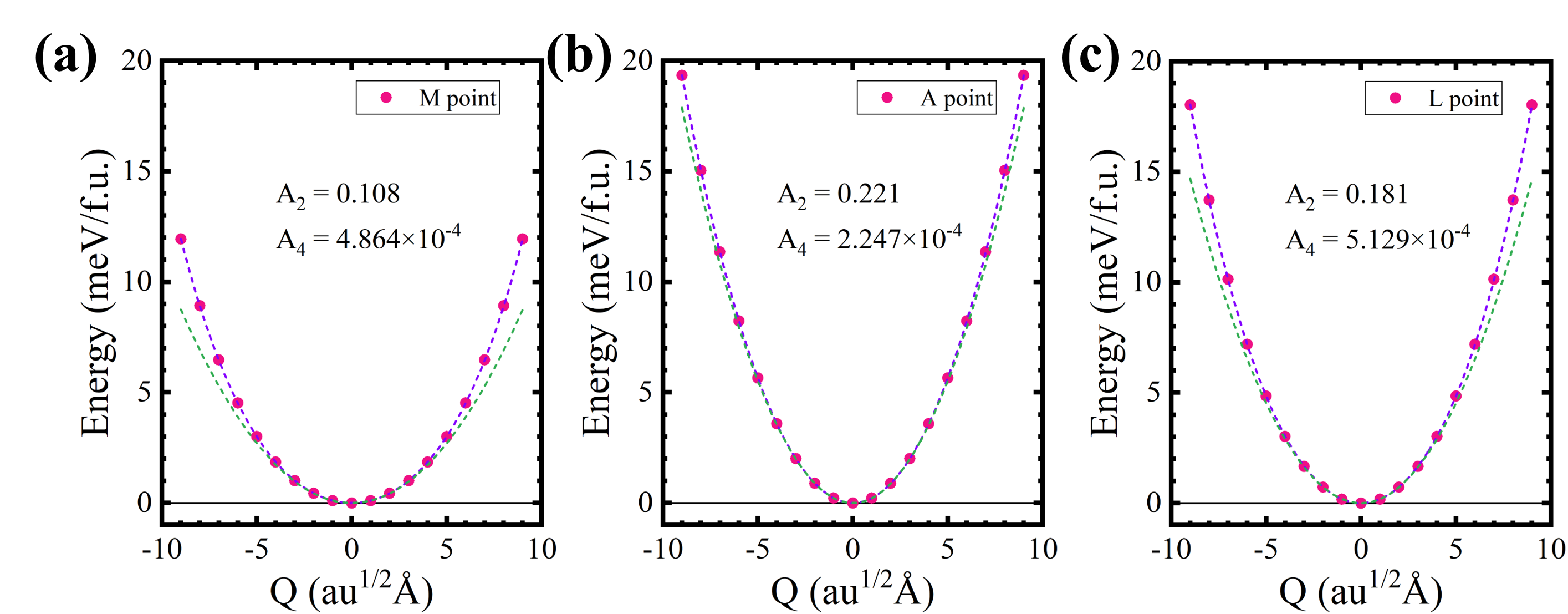


**Fig. S7** The potential energy surface corresponding to the low-lying transverse acoustic phonon modes at the Brillouin zone boundary M point **(a)**, A point **(b)**, L point **(c)**. The green dash line was fitted to 2-order, while the purple one was fitted to 4^th^-order, y = A_2_x^2^ + A_4_x^4^. When atoms move in larger magnitudes at elevated temperatures the potential energy surface deviates from the harmonic approximation and contains nonnegligible higher-order (for instance, quartic terms) contributions. This observation reveals that the phonon hardening essentially arises from the anharmonicity-induced phonon renormalization.


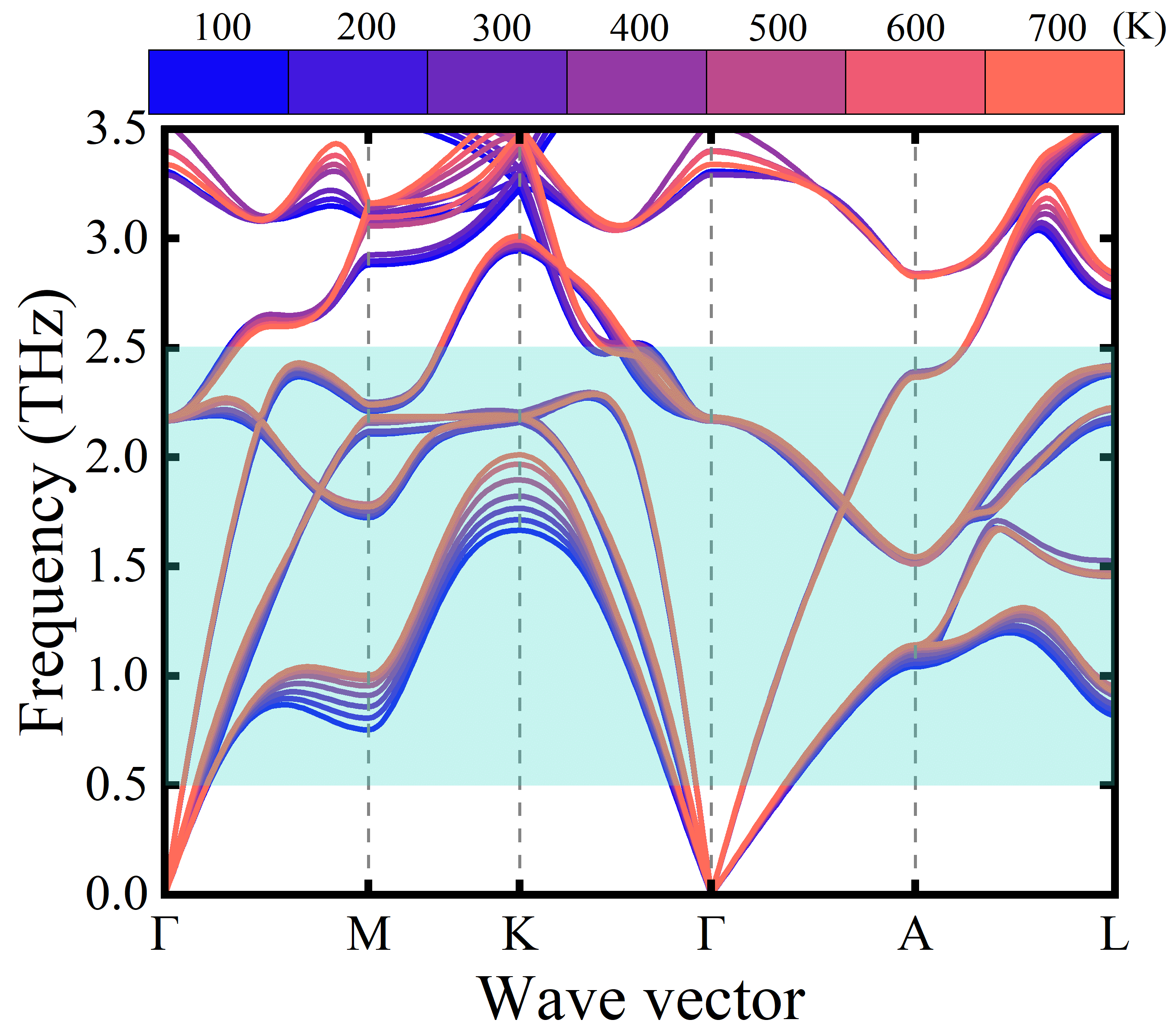


**Fig. S8** Calculated temperature-dependent phonon dispersions of Mg_3_Sb_2_ from *T* = 100 K to 700 K using the self-consistent phonon theory.


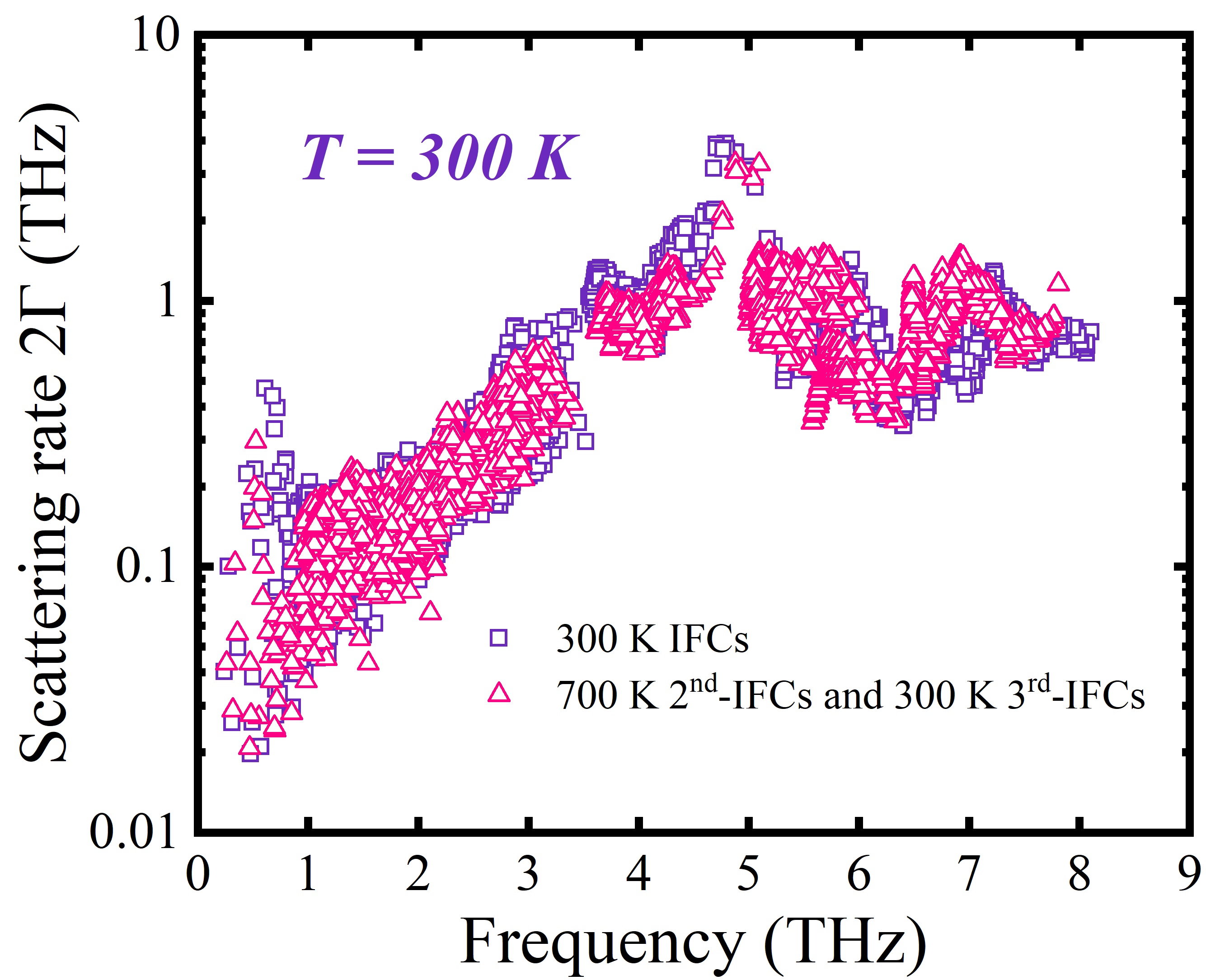


**Fig. S9** The scattering rate 2Γ with 300 K IFCs is compare to 2^nd^-order IFCs, substituted by 700 K, at *T* = 300 K.

**
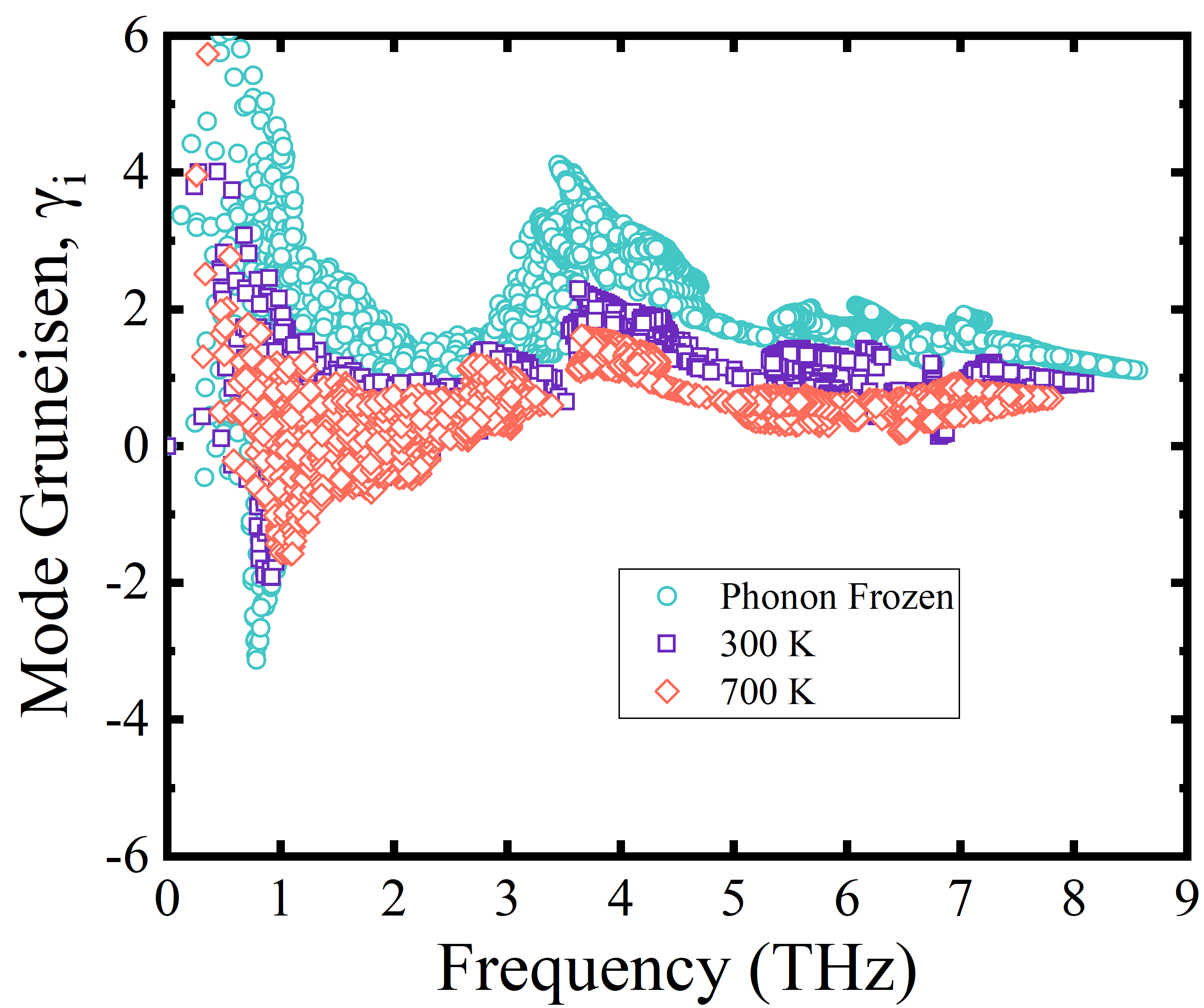
**

**Fig. S10** Frequency dependent the mode Grüneisen parameters using frozen phonon method[4], FTM at 300 K and 700 K.

**
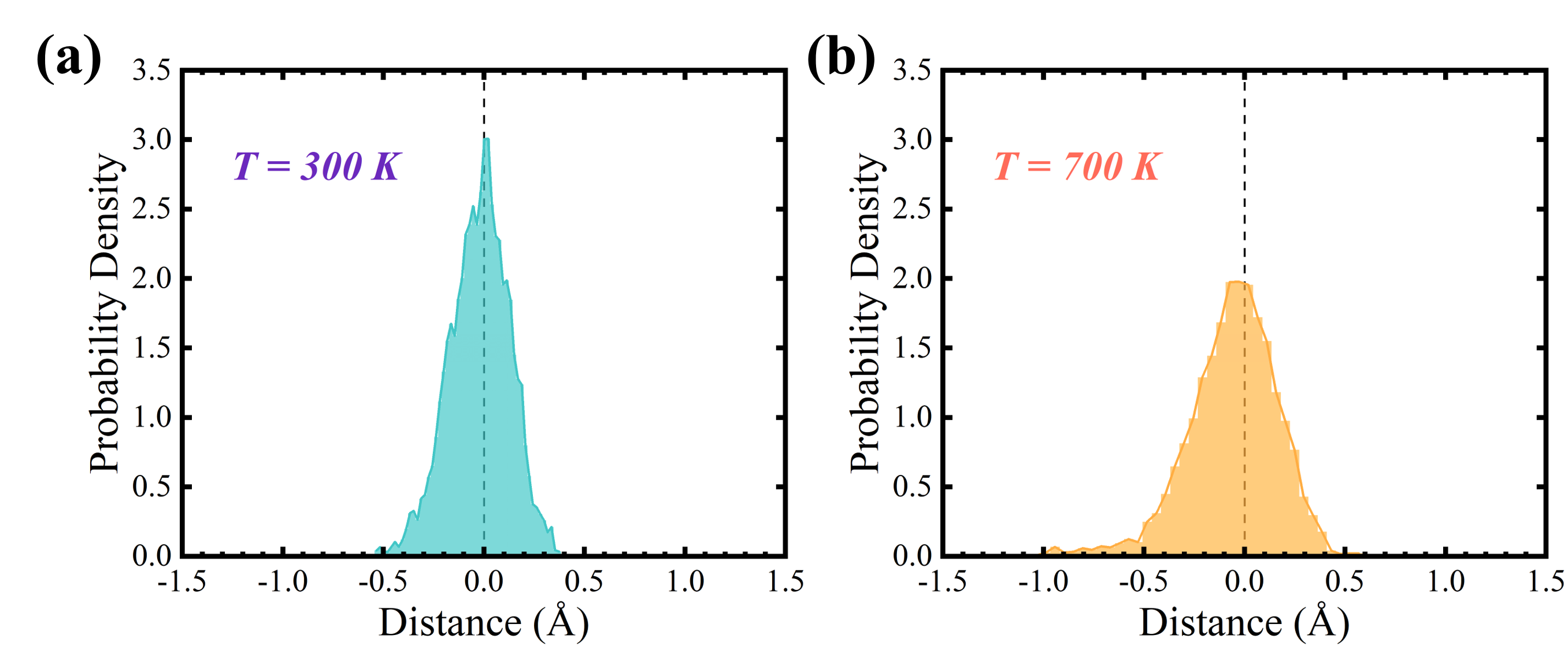
**

**Fig. S11** Probability density of MD trajectory deviating from equilibrium position at **(a)** 300 K and **(b)** 700 K along z direction.


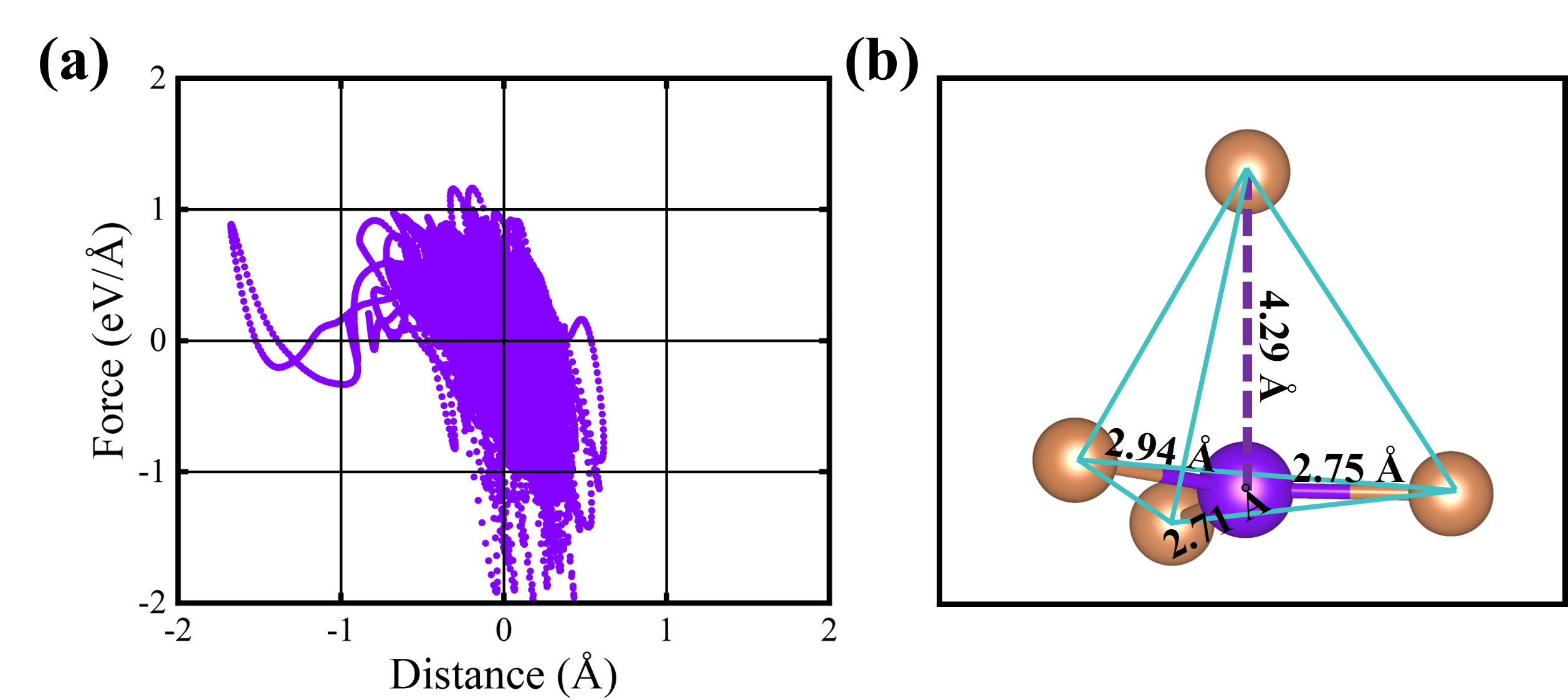


**Fig. S12 (a)** The force profile of Mg2 atom along z direction at 700 K, extracted from molecular dynamics simulation. **(b)** The local structure of Mg_3_Sb_2_ with the displacement -0.93 Å along z direction and the force -0.01 eV/Å.


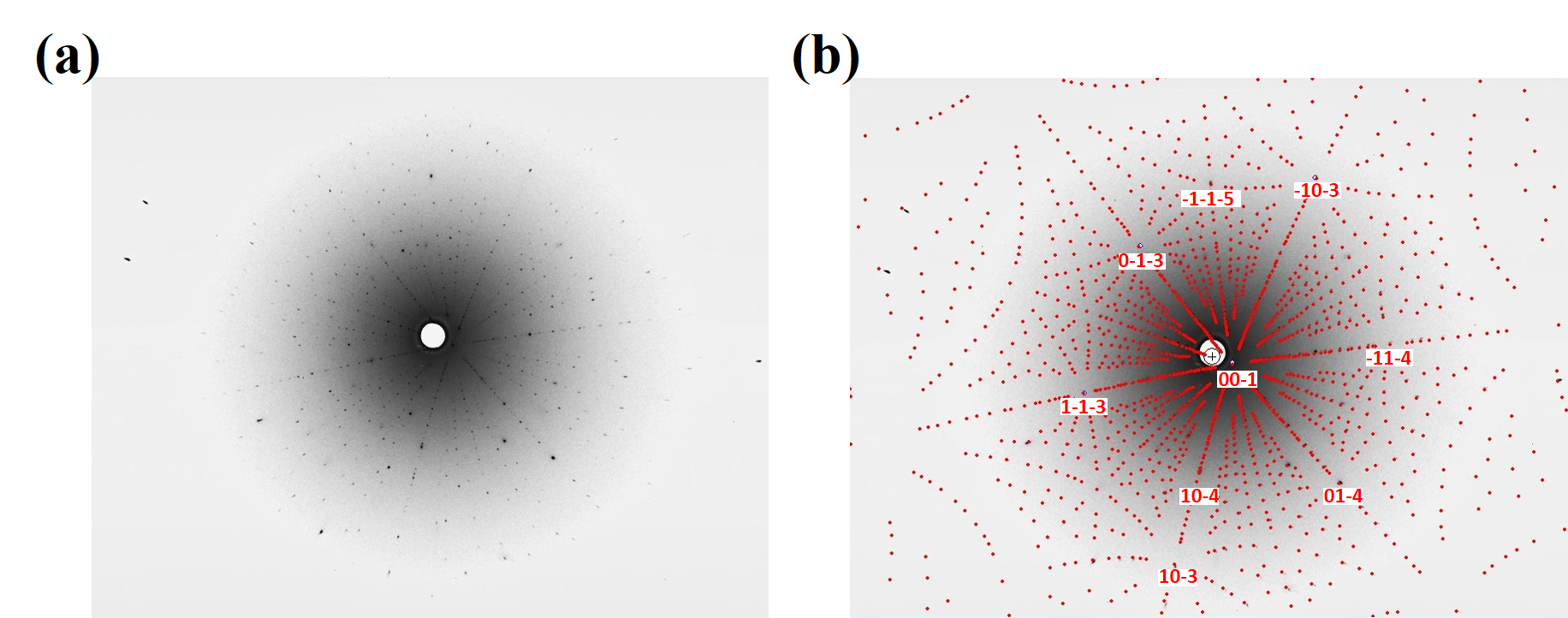


**Fig. S13 (a)** Laue diffraction pattern of the studied Mg_3_Sb_2_ single crystal and **(b)** the theoretically simulated pattern based on $\boldsymbol{P}\bar{\boldsymbol{3}}\boldsymbol{m}\boldsymbol{1}$ space group, matching well with the experimental data.

**Table S1** The calculated *κ_L_* along x and z directions at 300 K with temperature-dependent IFCs

| 2^nd^-order IFCs | 3^rd^-order IFCs | *κ_xx_* (W m^-1^ K^-1^) | *κ_zz_* (W m^-1^ K^-1^) |
| --- | --- | --- | --- |
| 300 K | 300 K | 2.50 | 2.05 |
| 700 K | 700 K | 3.83 | 3.15 |
| 300 K | 700 K | 4.05 | 3.21 |
| 700 K | 300 K | 2.36 | 1.97 |

## References

[1] L. Song, J. Zhang, and B. B. Iversen, “Simultaneous improvement of power factor and thermal conductivity via Ag doping in p-type Mg_3_Sb_2_ thermoelectric materials,” *Journal of Materials Chemistry A*, vol. 5, no. 10, pp. 4932-4939, 2017.

[2] J. Shuai, Y. Wang, H. S. Kim et al., “Thermoelectric properties of Na-doped Zintl compound: Mg_3-x_Na_x_Sb_2_,” *Acta Materialia*, vol. 93, pp. 187-193, 2015.

[3] A. Bhardwaj and D. K. Misra, “Enhancing thermoelectric properties of a p-type Mg_3_Sb_2_-based Zintl phase compound by Pb substitution in the anionic framework,” *RSC Advances*, vol. 4, no. 65, pp. 34552-34560, 2014.

[4] W. Li, J. Carrete, N. A. Katcho et al., “ShengBTE: A solver of the Boltzmann transport equation for phonons,” *Computer Physics Communications*, vol. 185, no. 6, pp. 1747-1758, 2014.
