# Supplementary figures and images for: Violation of the T−1 Relationship in the Lattice Thermal Conductivity of Mg3Sb2 with Locally Asymmetric Vibrations
Source: Research (Wash D C). 2020 Nov 30;2020:4589786. doi: 10.34133/2020/4589786 (PMC7877392; doi:10.34133/2020/4589786)

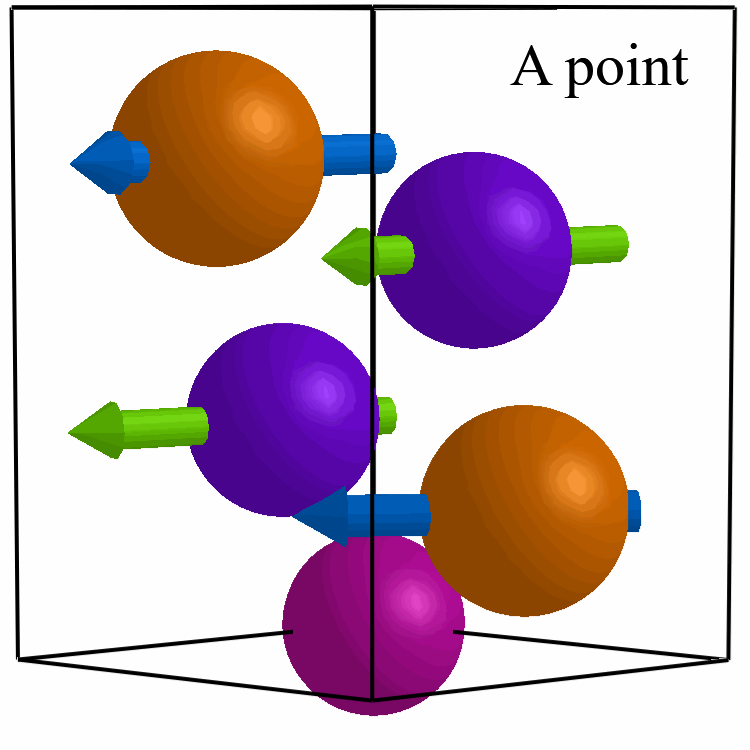

Supplement: Supplementary Materials — Fig. S1: experimental thermal conductivity data of polycrystalline samples. Fig. S2: phonon group velocity along three directions for Mg3Sb2 (a) at 300 K with the finite temperature method and (b) with the frozen phonon method. Fig. S3: (a) the temperature-dependent volumes of the primitive cell of Mg3Sb2 obtained by the high-temperature X-ray diffraction method, compared to those obtained using frozen phonon and AIMD calculation methods. (b) Lattice parameters are measured by high-temperature X-ray diffraction using Mg3Sb2 polycrystalline powders covering the range of 300 K to 700 K. Fig. S4: cumulative κL versus phonon mean free path for Mg3Sb2 at 300 K and 700 K. Fig. S5: the whole phonon dispersions of Mg3Sb2 at 300 K and 700 K. Fig. S6: calculated temperature-dependent phonon dispersions of Mg3Sb2 from T = 100 K to 700 K, only considering the lattice thermal expansion. Fig. S7: the potential energy surface corresponding to the low-lying transverse acoustic phonon modes at the Brillouin zone boundary M point (a), A point (b), and L point (c). Fig. S8: calculated temperature-dependent phonon dispersions of Mg3Sb2 from T = 100 K to 700 K using the self-consistent phonon theory. Fig. S9: the scattering rate 2Γ with 300 K IFCs is compared to 2nd-order IFCs, substituted by 700 K, at T = 300 K. Fig. S10: frequency-dependent mode Grüneisen parameters using the frozen phonon method, FTM at 300 K and 700 K. Fig. S11: probability density of MD trajectory deviating from equilibrium position at (a) 300 K and (b) 700 K along the z direction. Fig. S12: (a) the force profile of Mg2 atom along the z direction at 700 K, extracted from molecular dynamics simulation. (b) The local structure of Mg3Sb2 with the displacement -0.93 Å along the z direction and the force -0.01 eV/Å. Fig. S13: (a) Laue diffraction pattern of the studied Mg3Sb2 single crystal and (b) the theoretically simulated pattern based on P3¯m1 space group, matching well with the experimental data. Table S1: the cal [file 4589786.f1.zip › A.gif]

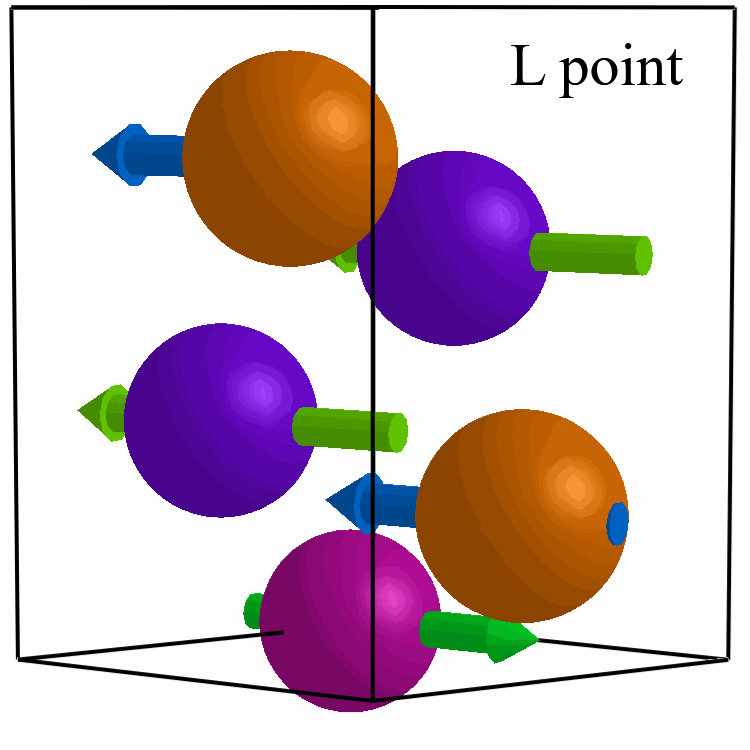

Supplement: Supplementary Materials — Fig. S1: experimental thermal conductivity data of polycrystalline samples. Fig. S2: phonon group velocity along three directions for Mg3Sb2 (a) at 300 K with the finite temperature method and (b) with the frozen phonon method. Fig. S3: (a) the temperature-dependent volumes of the primitive cell of Mg3Sb2 obtained by the high-temperature X-ray diffraction method, compared to those obtained using frozen phonon and AIMD calculation methods. (b) Lattice parameters are measured by high-temperature X-ray diffraction using Mg3Sb2 polycrystalline powders covering the range of 300 K to 700 K. Fig. S4: cumulative κL versus phonon mean free path for Mg3Sb2 at 300 K and 700 K. Fig. S5: the whole phonon dispersions of Mg3Sb2 at 300 K and 700 K. Fig. S6: calculated temperature-dependent phonon dispersions of Mg3Sb2 from T = 100 K to 700 K, only considering the lattice thermal expansion. Fig. S7: the potential energy surface corresponding to the low-lying transverse acoustic phonon modes at the Brillouin zone boundary M point (a), A point (b), and L point (c). Fig. S8: calculated temperature-dependent phonon dispersions of Mg3Sb2 from T = 100 K to 700 K using the self-consistent phonon theory. Fig. S9: the scattering rate 2Γ with 300 K IFCs is compared to 2nd-order IFCs, substituted by 700 K, at T = 300 K. Fig. S10: frequency-dependent mode Grüneisen parameters using the frozen phonon method, FTM at 300 K and 700 K. Fig. S11: probability density of MD trajectory deviating from equilibrium position at (a) 300 K and (b) 700 K along the z direction. Fig. S12: (a) the force profile of Mg2 atom along the z direction at 700 K, extracted from molecular dynamics simulation. (b) The local structure of Mg3Sb2 with the displacement -0.93 Å along the z direction and the force -0.01 eV/Å. Fig. S13: (a) Laue diffraction pattern of the studied Mg3Sb2 single crystal and (b) the theoretically simulated pattern based on P3¯m1 space group, matching well with the experimental data. Table S1: the cal [file 4589786.f1.zip › L.gif]

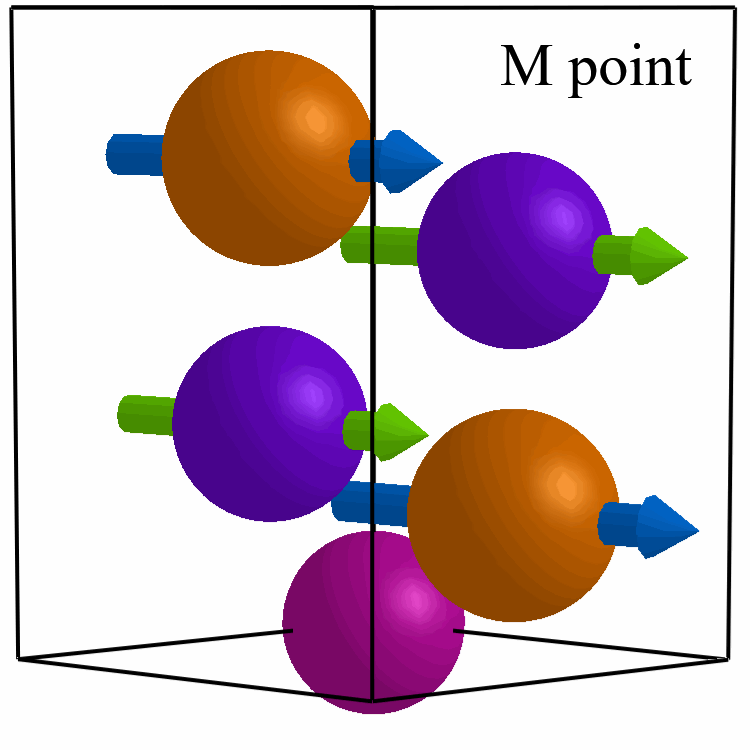

Supplement: Supplementary Materials — Fig. S1: experimental thermal conductivity data of polycrystalline samples. Fig. S2: phonon group velocity along three directions for Mg3Sb2 (a) at 300 K with the finite temperature method and (b) with the frozen phonon method. Fig. S3: (a) the temperature-dependent volumes of the primitive cell of Mg3Sb2 obtained by the high-temperature X-ray diffraction method, compared to those obtained using frozen phonon and AIMD calculation methods. (b) Lattice parameters are measured by high-temperature X-ray diffraction using Mg3Sb2 polycrystalline powders covering the range of 300 K to 700 K. Fig. S4: cumulative κL versus phonon mean free path for Mg3Sb2 at 300 K and 700 K. Fig. S5: the whole phonon dispersions of Mg3Sb2 at 300 K and 700 K. Fig. S6: calculated temperature-dependent phonon dispersions of Mg3Sb2 from T = 100 K to 700 K, only considering the lattice thermal expansion. Fig. S7: the potential energy surface corresponding to the low-lying transverse acoustic phonon modes at the Brillouin zone boundary M point (a), A point (b), and L point (c). Fig. S8: calculated temperature-dependent phonon dispersions of Mg3Sb2 from T = 100 K to 700 K using the self-consistent phonon theory. Fig. S9: the scattering rate 2Γ with 300 K IFCs is compared to 2nd-order IFCs, substituted by 700 K, at T = 300 K. Fig. S10: frequency-dependent mode Grüneisen parameters using the frozen phonon method, FTM at 300 K and 700 K. Fig. S11: probability density of MD trajectory deviating from equilibrium position at (a) 300 K and (b) 700 K along the z direction. Fig. S12: (a) the force profile of Mg2 atom along the z direction at 700 K, extracted from molecular dynamics simulation. (b) The local structure of Mg3Sb2 with the displacement -0.93 Å along the z direction and the force -0.01 eV/Å. Fig. S13: (a) Laue diffraction pattern of the studied Mg3Sb2 single crystal and (b) the theoretically simulated pattern based on P3¯m1 space group, matching well with the experimental data. Table S1: the cal [file 4589786.f1.zip › M.gif]
